# Animal Research Regulation: Improving Decision-Making and Adopting a Transparent System to Address Concerns around Approval Rate of Experiments

**DOI:** 10.3390/ani14060846

**Published:** 2024-03-09

**Authors:** David Mawufemor Azilagbetor, David Shaw, Bernice Simone Elger

**Affiliations:** 1 Institute for Biomedical Ethics, University of Basel, 4056 Basel, Switzerland; david.shaw@unibas.ch (D.S.); b.elger@unibas.ch (B.S.E.); 2 Care and Public Health Research Institute, Universiteit Maastricht, 6229 ER Maastricht, The Netherlands; 3 Center for Legal Medicine, Unit for Health Law and Humanitarian Medicine, University of Geneva, 1205 Geneva, Switzerland

**Keywords:** animal experimentation, animal research oversight, animal experimentation committees, harm–benefit analysis, 3R, approval rate, societal concerns

## Abstract

**Simple Summary:**

Scientific experiments that use animals are strictly regulated, mainly as a result of public concerns about animal testing. Examination of the regulation of animal experiments shows that most of the experiments submitted for authorization are approved. But what could be the reasons for this high approval? Since public understanding is needed in order to reach a common agreement that will shape animal research policy, it is important to have a transparent discussion about what leads to the frequent approval of animal experiments. The aim of this article is to discuss why frequent approval of experiments is a problem: the regulatory process has been put in place to authorize only acceptable experiments. Even though some experiments, such as cosmetic testing on animals, are prohibited in many countries irrespective of harm–benefit ratios, most types of animal experiments are approved. Further, we explain some possible genuine reasons for frequent approval, but at the same time, we also discuss how the regulatory process could be leading to the approval of unacceptable studies. To ensure transparency, gain public trust, and improve the understanding of the regulation of animal testing, we propose that regulators publish their experiment evaluations on an accessible online platform, explaining how they reached their decisions.

**Abstract:**

The use of nonhuman animals in biomedical research is regulated under stringent conditions, not only in response to societal attitudes towards animal experimentation but also because ethical responsibility in scientific research requires researchers and veterinarians to be more invested and aim to improve the welfare of animals used for experiments. Analyses of animal research oversight reveal the frequent approval of experiments, and the approval of some experiments has raised and continues to raise public concerns. Societal compliance is required for a consensus-based approach to animal research policy, prompting the need to have transparent discussions about oversight and the frequency of approvals. We discuss how frequent approval may be perceived and why it seems problematic from a societal perspective: the regulatory process exists to approve only legitimate experiments. Although some experiments remain unacceptable irrespective of their harm–benefit ratios, almost all experiments are approved. We explain some possible legitimate reasons for frequent approval and how the review process could be leading to the approval of illegitimate studies. To ensure transparency and improve public trust and understanding of oversight, we propose the adoption of a platform to inform society about how unethical experiments are screened out.

## 1. Introduction

The use of nonhuman animals (henceforth animals) as models in biomedical research and regulatory testing raises many concerns related to ethics [1,2] and scientific rigor [3], and the current consensus regarding this use is based on the principle that only animal experiments with foreseeable benefits outweighing harm caused to the experimental animals should be allowed [4]. For this reason, animal research in many countries is regulated under strict regulatory policies, and any research project involving animals requires an ethical assessment followed by a license to proceed. When submitting a research proposal for authorization, researchers state the objectives, benefits, and scientific justifications of their study [5]. They also provide information regarding steps taken to address animal welfare concerns, addressing points such as the housing conditions of animals and the use of anesthesia and analgesia for experimental techniques, as well as other modulating factors—factors that could mitigate and/or aggravate harm (for example, type and size of caging) [6]. Information regarding the training and expertise of researchers working on the project [5], including their possession of skills in the use of hazardous agents and in recognizing behavioral signs that might indicate severe pain leading to endpoints, is also given.

Responsible oversight authorities carrying out project assessments have different designations in different jurisdictive contexts, though their primary duties are similar [5,7,8,9]. In this essay, they will be referred to generally as *oversight bodies* or *committees* and specifically as *animal experimentation commissions (AECs)*.

The functioning of these oversight bodies as well as animal research regulatory procedures in general have been rigorously investigated over the past decades, and one of the major issues that has frequently been raised is the approval rate of animal research projects. It has been observed that a vast majority of animal experiments, indeed almost all, submitted for ethical review are approved and licensed to proceed [10] either directly or after significant modifications [9,10,11,12,13]. In Europe, for example, data on percentage rejections presented show that in several countries, including the UK, France, Germany, Spain, Poland, and Denmark, all submissions made for evaluation have been approved (i.e., no applications were rejected) [13]. The studies on oversight bodies focus more on understanding their functioning and adherence to ethical and legal obligations, and not enough explanation has been given for the observed frequent approval of projects. It is worth noting that the frequency of project approvals is a considerable criterion for the output of AECs and thus says a lot about their effective functioning. Rightly, it has been argued that the high rate of approval calls into question the purpose and function of oversight bodies [14].

The main objective of this article is to describe and analyze the process of animal research oversight and inform oversight bodies of ways to improve decision-making to legitimize decision outcomes. In line with this objective, we will start by discussing the ethical review process and the criteria considered. Then, we will describe how the acceptability or unacceptability of experiments is determined. Further, we will focus on ethical and societal concerns regarding the frequent approval of animal experiments, leading to the discussion of possible legitimate reasons for low rejection and high approval rates. In light of these legitimate reasons for frequent approval, we will also discuss why the current review process could still illegitimately lead to high approval. Based on the need to improve the decision-making process of oversight bodies, we will discuss how the oversight process should be approached to ensure the legitimacy and ethical justification of decision outcomes. Finally, by contributing to the drive for more transparent discourse on animal research regulation in society to improve public trust and understanding of animal research oversight, we will propose the adoption of a channel through which society can be informed about how illegitimate experiments are identified and screened out.

By adding to discussions around the legitimacy of animal experiment evaluations and approvals, this essay will educate the public about animal research oversight and inform oversight bodies of ways to improve their decision-making to legitimize decision outcomes. More generally, it will inform animal research policy based on transparency to advance public trust and a consensus-based approach to animal use in science.

## 2. The Ethical Review Process

Having a fair and acceptable constitution of oversight bodies is required to reflect the suggestion that ethical review procedures should adopt a democratic approach where different views from society are expressed [15]. Oversight bodies are therefore constituted of members with various competencies, perspectives, and moral outlooks. Members usually include scientists, ethicists, lawyers, laboratory animal specialists, representatives from animal protection organizations, members of the lay public, and so on. A committee of people with diverse opinions creates room for dialogue where the members, starting with differing initial positions, express their conflicting views and interests, but down the line, on behalf of society, jointly draw boundaries around what is and is not acceptable as far as animal experimentation is concerned [15,16] and, at the end, accept or reject experiments seeking the use of animals on a case-by-case basis. In their assessments regarding the approval or rejection of experiments, they make sure that the principle of the 3Rs (*replacement, reduction, and refinement*) is maximized, and they also carry out a *harm–benefit analysis (HBA)* to ensure that the benefits to be gained from the experiments outweigh the harm to be caused to the experimental animals.

### 2.1. The 3Rs

The 3Rs, three popular principles guiding animal experimentation, were originally conceived by Russell and Burch [17] in their book, *The Principles of Humane Experimental Technique*. The first of these principles requires that before an experiment is planned to be carried out on animals, non-animal alternatives should be sought, i.e., replacement.

If no robust alternative methods in accordance with the current state of knowledge exist and animals are still required for the experiment, efforts should be made to reasonably reduce the number of animals to be used without compromising scientific quality, i.e., reduction. Further, animal welfare measures should be considered in the experimental procedures to reduce animal pain and distress, i.e., refinement.

Researchers are required to fully apply these principles when planning their experiments, and they must explain their implementation in their projects when making a submission for ethics approval. For example, the use of non-mammalian animals such as fish, insects and nematodes, which could be considered less sentient by society [18], to study the basic biology of health conditions [19] could be considered an application of the 3Rs. Also, scientific and technological advancements have led to improved implementation of the 3Rs. As a result, the concept of the 3Rs has been evolving over the years, and significant aspects of the three Rs have been in continuous improvement. For example, partial and full replacement of animals in investigating some research questions has been said to be achieved through innovative technologies such as lab-on-chip technology (which enables the modelling of cancerous lymphatic vessels [20]), the use of organoids (in researching tissue-specific diseases [21,22]), and other in vitro technologies (involving the culture of primary human cells [23,24]). Modern technologies offer opportunities to reduce animal usage by providing means of obtaining more objective and measurable data for research [25]. Also, contemporary approaches to refinement involve the use of novel in vivo technologies that reduce the pain or distress undergone by experimental animals and provide means of delivering improvements in animal housing, care, handling, and use [26].

While the 3Rs have worked and continue to serve the animal research community well, other Rs have also been proposed towards the advancement of animal research ethics. This is a result of the progression of awareness and understanding of animal research ethics by the animal research community, resulting in new developments and requirements to reflect culture, values and societal perspectives. In making the 3Rs more comprehensive, Brink and Lewis [27], for instance, proposed extra Rs, presenting a total of 12 Rs (the 12Rs framework) as a guide for animal research ethics to reflect the multitude of factors to consider in animal experimentation. These additional concepts make the 3Rs robust enough to accommodate the other important factors (including the responsibility of animal researchers, reproducibility in research, relevance of an animal experiment, respect for animals, etc.) involved in the complexity of animal research ethics.

After considering the 3Rs (and conducting an HBA in some contexts like Switzerland) at the very beginning of research planning and experimental design, the animal research proposal is submitted to oversight authorities for verification of the application of the 3Rs, followed by an HBA to make a final decision.

### 2.2. Harm–Benefit Analysis (HBA)

In general, cost/risk–benefit analysis is a common decision theory applied in several domains as a decision aid. In animal research, this concept is now widely referred to as ‘harm–benefit analysis’ [28,29,30]. This moral concept originates from utilitarianism as conceived by Jeremy Bentham [31]. According to utilitarianism, the ethics of an action should be controlled by the notion of the greatest benefit for the greatest number involved. Utilitarian ethical theory, also referred to as consequentialism [32,33], means the ethical justification of an intended action will be determined by the consequences of the action and how useful the consequences will be in making society better [34,35,36]. In the context of animal research, the employment of animals in scientific endeavors should be condoned and guided on the condition that the benefits acquired from such employment exceed the harm caused to the animal study subjects [37]. In the words of Bentham [38] when he wrote to the editor of the *Morning Chronicle*:

*‘I never have seen, nor ever can see, any objection to the putting of dogs and other inferior animals to pain, in the way of medical experiment, when that experiment has a determinate object, beneficial to mankind, accompanied with a fair prospect of the accomplishment of it. But I have a decided and insuperable objection to the putting of them to pain without any such view.’*

Through this theory, it is held that animal experimentation could be justified by the premise that the end result of such an experiment justifies the means of the animals used in the experiment. This makes it clear that animal suffering is only justified if benefit arises from it.

Currently, animal research—in spite of growing discussions within many societies—remains acceptable, but to make sure that the practice is ethically acceptable and in line with the legal and moral status of animals, animal research must satisfy the utilitarian condition before it is decided if the experiment should proceed or not. In the process of HBA, harm to animals is balanced against benefits (mainly benefits to humans) that will accrue from the experiment [10,39]. This exercise requires that the possible harm to animals and the benefits of the experiment be adequately evaluated to inform the final decision. A report compiled from the review of HBA by the UK Animals in Science Committee (ASC) [40,41] provides a detailed discussion of HBA in practice.

## 3. The (Un)Acceptability of an Animal Experiment

As stated above, for a research project to be allowed to use animals, the harm caused to the animals involved in the research should be balanced against the expected benefits of the research. Depending on the results of this analysis, the project will either be approved or denied. In practice, reaching this decision is not an easy task, and much has been written describing the advantages and disadvantages of HBA [36], how it tends to limit scientific advancement [42], and how it might not improve laboratory animal welfare [43].

The scientific and societal benefits of an animal research project are assessed during HBA. Scientific benefit, usually resulting from basic research, concerns ground-breaking knowledge with scientific insight where an impact should be within reach. In proposing a matrix to support the measurement and balancing of harm and benefits, Bout and colleagues [39] defend the position that for ‘basic research with great scientific benefit but no reasonable expectation of near-term (e.g., within 20 years) applicability, no more than moderate harm is acceptable’. This suggestion stresses the importance of the applicability of animal research results in the near term as a complement to the actual potential of the results in scientific advancement. Societal benefits are rather closer to practical implementation and include benefits regarding the application of knowledge to address societal problems including human and environmental health issues [39]. To weigh these benefits, factors such as the seriousness of the human disease in question and the number of patients it affects could be considered [40]. How readily the benefits of an animal experiment would be available within a short time might also play a role in the HBA. An example is the use of non-human primates during the COVID-19 pandemic to understand the pathogenesis of the severe acute respiratory syndrome coronavirus 2 (SARS-CoV-2) to develop vaccines and therapies for the coronavirus disease [44,45,46], even though research with non-human primates is highly controversial and challenging [47,48]. (The number of people affected by the virus during the pandemic might have made it possible to recognize and quantify the benefits, leading to a more informed HBA decision, an exercise recommended by experts in the conduct of HBA [49]).

The likelihood of benefits depends on the robustness of the project’s experimental design. A poorly designed research protocol and experiment is unlikely going to give the expected benefits. Therefore, the scientific validity of an experimental protocol might also be a significant consideration in HBA [6,28]. Further, evidence of the success of proposed experiments could be required when evaluating them before their authorization, which could depend largely on the persuasiveness, confidence, and track record of the researchers [40,49,50].

The minimization of harm—refinement—is achieved by the improvement of experimental design or by using less harmful techniques; the use of pain relief methods such as anesthesia and analgesia; and the improvement of transport, housing, and care conditions of the animals [28]. In this sense, the level of harm left after all these refinements is what will finally be balanced against the benefits. According to what is expected from this assessment process, an experiment whose foreseeable benefits outweigh animal harm and within which issues regarding animal welfare have adequately been addressed is acceptable and should be approved. However, an experiment whose HBA results fall short of these outcomes is unacceptable and should be rejected.

## 4. Approval of Animal Experiments and Ethical and Societal Concerns

Animal research is contentious, and the ethical assessment of animal research projects plays a crucial role in ensuring that animals are not only used in the most humane manner but also in and for the right experiments. As Kalman and colleagues [29] put it, ‘society expects that bodies such as ethical committees will take corporate social responsibility by acting as watchdogs for animal experiments […]’. Through a regulatory process that serves as a system to sieve out illegitimate experiments, all stakeholders involved—the research community and society—would be assured of rigorous scrutiny of animal experiments. This perspective is in line with political responses in which policymakers assure society that a system exists to ensure that only legitimate animal experiments are conducted [12]. As a consequence of this societal consensus regarding the use of animals in research, the current regulatory procedure may be perceived by some as one that brings objective regulation of animal use in research into disrepute, especially when almost all experiments are eventually accepted and conducted.

There may be some explanations as to why some members of the public could view the approval of almost all experiments as questionable. From a societal perspective, there are some experiments for which animal suffering, even at the lowest harm severity level, would not be worth the benefit, irrespective of how robust the experimental procedures are. Let us take, for instance, cosmetic testing on animals, which is totally prohibited in the European Union, Australia, and several other countries [51]. This prohibition, which resulted from societal objection, reveals that testing finished cosmetic products and, to some extent, cosmetic product ingredients on animals is frowned upon by society, irrespective of how low the harm to the animals might be or how impactful the benefits of the cosmetic product will be. From this view, according to a large majority of the public, no amount of benefit to be gained from cosmetic experimentation will ever outweigh animal harm, and in this case, animal lives or welfare are categorically considered not worth exploiting for cosmetic production benefits. The approval of almost every animal experiment subjected to ethical review, therefore, seems problematic, especially if society is not sure and also if it is not assured that the review process through HBA aligns with current societal consensus and that all approved animal experiments ultimately address legitimate societal interests.

It is worth noting that the observation of frequent approval is not restricted to animal research oversight alone; it could also be observed in human subject research oversight, where almost all projects are approved [52]. That being the case, high approval in animal research seems to be more problematic because animals would have no opportunity to consent to their participation in the experiments, whereas human subjects would have another opportunity to give consent or decline their participation, even in studies considered ‘high-risk’ [53]. For this reason, the vulnerability of animals makes high approval more delicate in the context of animal research oversight. In addition, from general observation, it could be possible that AECs have less public trust when it comes to ethical oversight as compared to human research ethics committees (RECs), and as such, the legitimacy of final decisions reached by and the accountability of the former appear to be more questionable than the latter.

Due to all these complexities, some level of responsibility is expected from oversight bodies and the current animal research review process as a whole. In this regard, decision outcomes and all experiments approved by AECs are expected to be justified based on these societal concerns and expectations.

## 5. Possible Legitimate Reasons for Low Rejection and High Approval Rate

One fact that remains is that approving almost all animal experiments does not necessarily mean that some or all of the approved projects are illegitimate. (Projects being illegitimate here mean projects that are not in accordance with the accepted standards or rules, and in this case, the 3Rs and HBA.) The high approval rate may be due to the fact that animal researchers are aware of the requirements their projects need to meet in order to have their experiments accepted [5], and they submit their applications accordingly. For that matter, only acceptable projects are submitted to AECs. In Switzerland, it is understood that the oversight process requires all animal researchers to argue why their experiments should be accepted. They are required to conduct their own HBA and weighing of interests before submitting applications to AECs [54]. In theory, it could be said that when researchers proceed to submit their applications to AECs, the HBA they conducted prior to this submission might have been positive, and the experiments might have seemed acceptable in their own eyes, so they are less likely to submit unacceptable projects to AECs. This, to some extent, could explain why AECs end up approving almost all the experiments they receive for review.

Furthermore, most animal experiments are funded in that, prior to funding, they go through review, and there is a high possibility that there has been a strong filter to take unacceptable ones out before the rest reach AECs. In addition, in many research institutions, animal researchers are advised to collaborate with animal care and welfare officers when planning animal experiments. As a result, animal harm and welfare issues would have been addressed from the very onset, which would make the projects more acceptable before they arrived at AECs. Additionally, animal experiments are costly and require substantial human and material resources. The regulatory and AEC procedures also require considerable time and attention from the researchers. All these reasons could make it less likely that a questionable experiment that would likely not be approved would be submitted.

Moreover, the strict attention oversight bodies give to refinement as a major factor in making decisions could play a role in why most of the experiments are approved. In the review of experimental protocols, some of the researchers are asked, usually in a back-and-forth manner, to modify their experiments until animal welfare issues are addressed and animal harm is reduced to an acceptable minimum before experiments are approved. This refinement process could be justified by the fact that animal welfare in experiments is the main worry of society and, for that matter, is always at the heart of the ethical review process. Moreover, animal research is only permissible if no alternatives exist, and it could be argued that AECs do not see experiments in which animals are replaced following the implementation of the replacement principle of the 3Rs, since a proposal would not be put to them. It would be expected that in vitro investigations, for instance, would have preceded a submission, and AEC procedures would focus more on these preceding works and refinement. A review demonstrates that AECs seem to have a stronger tradition of focusing on harm [28]. Further, a critical analysis of animal research projects approved after AECs requested modifications finds that the majority of the aspects modified fall under *refinement* [9]. Thus, generally, AECs try to resolve harm and welfare issues, which tend to lower animal harm, making projects more acceptable than unacceptable when they conduct HBAs.

The complication of this observed frequent approval is that, even for the handful of experiments that may be rejected when harm is inadequately addressed and benefits are not well documented, researchers can still redesign and resubmit them to AECs for review. These experiments, in their new forms, may become more legitimate and may finally be approved. This argument seems to spare us the need to worry so much about how experiments are frequently approved; however, the assurance it gives would only have a major impact if there were no other explicable phenomena that might influence the decision-making process, leading to illegitimate frequent approval. We discuss these in the next section.

## 6. Could the Current Review Process Still Illegitimately Lead to High Approval?

As described above, in a transparent system of animal research regulation where requirements and all aspects of animal research, including technical and animal welfare issues, are well elaborated and addressed before the projects reach AECs, decisions may always be favorable towards animal research. However, these high approval rates may be questionable if there still remain coexisting concerns regarding the quality of the oversight process, which leads to high approval.

It has been discussed whether, in general, the outcome of committee reviews could be influenced by how the committees are constituted and by how discussions progress. Due to their varying backgrounds, committee members focus on different aspects (either harm or benefits) of animal experiments, a result one would expect when one forms a group with diverse stakeholders. That being said, applications being approved, at least after modification, has been seen as an approval bias brought on by committees with a preponderance of members (mainly scientists) who have a stake in research [12,55]. Several studies show that some expert committee members, like scientists, dominate discussions, while other members, like lay public representatives, rarely make contributions [56,57]. It is explained that this inadequate contribution to debates by community members is due to the fact that scientists outnumber them [58], resulting in their reservations leading to their inability to express their opinions. (It is worth noting that this might not be the case in a context, like Australia, where scientists do not outnumber lay persons). In contention with the argument that scientists outnumber community members on oversight committees, research shows again that even in contexts where there is equal representation of all stakeholders, scientists may still dominate the discussions [59]. It is therefore noted that regardless of whether they are in the majority or not, scientists’ knowledge of science, a crucial area of knowledge for the committee’s discussions which is however typically out of the reach of non-scientists, would likely always give them an advantage [15], leading to high approval. Can we be sure that this imbalance does not go in favor of all projects being approved? This is not to say that only scientists may be inclined toward a particular aspect of the experiment because community members are also liable to this bias, and it has been observed that they are also more inclined to talk about animal suffering and distress than scientists [56]. However, since a high approval rate tends to go in favor of scientists’ position, they would normally be held responsible for their role in the decision-making process.

Another situation in which ethical assessment would lead to illegitimate high approval is when animal welfare issues are not adequately addressed. Sometimes, identifying, understanding, quantifying, and mitigating harm to experimental animals might not always be evident. Arnason and Clausen [60] described problems encountered by HBA in evaluating harm and benefits and balancing them against each other. With a particular focus on monkeys in neurological research, they highlighted the difficulty of harm evaluation, stating that the type of suffering that can be expected ‘only tells us what types of harm are possible and where to look for them, but it does not even start evaluating how much harm is involved or how to go about conducting such an evaluation’. In another instance, after analyzing biomedical research projects involving genetically modified (GM) animals that had been approved by AECs in Sweden, Nordgren and Röcklinsberg [61] discovered that ‘applications were often approved by the committees despite containing insufficient information regarding ethically relevant aspects, that the arguments for using GM animals were often unclear, and that some applicants indicated awareness of possible unintentional welfare effects attributable to genetic modification’. First and foremost, AECs need to at least have information on these aspects to inform legitimate decision-making. However, it appears that they may be approving experiments without them, or should it be said that they might be approving experiments without sufficient review? If mitigating harm in animal research is a top priority in HBA and ethical oversight in general, and animal welfare issues are ignored when approving experiments, the legitimacy of the high number of experiments approved could be questioned. Additionally, while current advancements in laboratory animal welfare science are made to address animal distress concerns in experiments, there are limitations to the practical implementation of tools in understanding and addressing pain and emotion [62]. If it is possible that there may be some uncertainty regarding the understanding and mitigation of some aspects of harm as they are truly being suffered by the animals [60], how can one be sure that, according to the HBA principle, the benefits of the high number of approved animal experiments outweigh the ‘hard to assess’ harm the animals actually suffer?

Sometimes, revision or modification of an experimental protocol may be necessary and, in the end, can make a project look more acceptable for a positive decision. The objective of this revision may be to improve the scientific quality of the experiment, which will increase the likelihood of the promised results and benefits [28]. As described above, a revision of the experimental technique in other instances may also be targeted at addressing animal welfare issues by choosing more humane techniques [9,11]. However, experimental protocols alone do not determine how acceptable an experiment is. With reference to the ethics of animal research, especially HBA, the probability and the magnitude of prospective benefits also need to play a significant and an equal role in the decisions. While these revisions may sometimes end up reducing animal harm, which is a good thing, the initial potential benefits may also not be augmented in parallel (and could even be diminished). At the end, these revisions may paint a picture of the benefits (which might have been considered lower from the beginning) outweighing harm, which will make the experiment more acceptable through the lens of HBA because animal harm is later considered lower than the expected benefits, leading to the acceptance of the experiment. To some extent, some might speculate that this way of approaching HBA and oversight in general makes decisions more inclined towards approvals rather than the rejection of unacceptable experiments.

## 7. Is There Room for Further Legitimatization of Decision-Making Processes and Outcomes?

The improvement of the functioning of AECs is a work in progress, and therefore, more efforts need to be made to make sure that the outcomes of reviews reflect the purpose envisioned for animal research oversight. Table 1 summarizes possible causes of illegitimate approval of experiments and how they can be mitigated. Many steps can be taken in this regard:

It is recommended that when committees make their decisions, they need to refer to past project reviews that have been accepted or rejected, including those beyond their own evaluations carried out by other committees operating under the same legal mandate, to ensure transparency [15]. We note here that results from the review of current experiments do not always have to be based on exact or similar results from preceding reviews, since the main objective here is to improve the review process and legitimize final decisions. AECs can, however, learn from errors from past reviews, especially in cases where retrospective review outcomes of approved projects exist. By taking this approach, AECs will gradually develop a nuanced understanding of where to draw the line between acceptable and unacceptable experiments by setting thresholds for how much suffering of laboratory animals is tolerable in order to achieve various sorts of benefits [15], and the legitimacy of approved experiments will be ensured.

Furthermore, having a level ground for balanced discussions is ideal and should be ensured. The observed imbalance in committee discussions [56,57,58] could be resolved by constituting committees with equal numbers of representatives from different fields and equal representation by non-expert members. As observed by Silverman et al. [56], even just by taking a particular position at the beginning of a discussion, the direction of deliberations may change in favor of scientists, since they are thought to make up the bulk of the presenters, contributing to their dominance of discussions. Committee leads should be conscious of these risks stemming from the unintended dominance of experts and address them by making sure that all members are given a chance to speak. Members of committees agree that a chairperson would play a crucial role in ensuring the smooth running of proceedings, ‘maintaining an open and respectful atmosphere where all views are accepted’ and also help the committee to ‘reach a consensus’ [58].

General agreement reached by consensus regarding final decisions should be prioritized over the *majority-carries-the-vote* type of decision-making. Final decisions are mainly democratic and based on votes cast by each member, where majority votes determine the fate of projects after group discussions. Even though a few individual members may still disagree with the vast majority, final decisions will reflect the majority’s stance. To thin down the line of disagreement among members on final decisions, committees must ensure that all members and their perspectives are taken along and included in deliberations to reach a mutual agreement before votes are cast. Any dissenting votes must also be recorded in committee minutes in the interest of transparency and for any future appeals. Admittedly, it might be difficult to have all committee members arrive at the same conclusion regarding benefits and animal harm, since scientists may tend to support and defend benefits while lay members may rather always be concerned with animal harm. However, we can still bring differing perspectives closer by ensuring that every member is convinced by the majority’s position after deliberations before votes are cast to reduce the level of disagreement, as some members may still remain unhappy when final decisions do not go in their favor. This, we suppose, will be of immense contribution to the actual legitimacy of approved projects.

Moreover, care must be taken when committees adopt an approach that could be referred to as ‘*modify for approval*’. Excessive modifications, as discussed above, may throw the purpose of HBA off balance, and the ethical acceptability of experiments may hardly be seen. It is believed that committees’ arguments on the approval of animal research almost exclusively center on specifics and technicalities rather than broader moral standards [39], and this may jeopardize the ethical acceptability of approved experiments. All aspects of experiments, both the technical aspects and the ethical concerns, should be addressed equally before making decisions. Indeed, refinement could fall within both areas of concern: the technical aspect of the experiment as well as a principle that addresses animal welfare. However, the point here is that efforts should be made to make sure that excessive refinement does make projects with perceived low benefits (from the very beginning) acceptable after significant modifications, since modification may not necessarily increase benefits but may rather decrease them.

## 8. Advancing Transparency of Animal Research Oversight and Decision Outcomes

The lack of open communication about animal research has not helped in addressing societal questions in order to gain societal approval for animal research. In the past few decades, openness to the public about animal research has been seen as an appropriate and most promising approach to addressing societal concerns. Openness about animal research could improve public backing, and scientists have been adopting this strategy to pursue public understanding of animal research and to gain the meritable trust and support of the public for their research [63]. Scientists are urged to communicate openly about the species of animals they are using for their experiments, the types of diseases they are investigating, and animal husbandry practices in their research laboratories [63,64]. Additionally, efforts are being made to open up animal research to the public. In the EU, for instance, the publication of non-technical summaries of approved experiments is a requirement [65]. This is to ensure that information on experiments using animals is publicly available to increase transparency.

These efforts are commendable; nevertheless, openness about animal research should not be left to only scientists conducting animal research or the research institutions where animal experiments are performed, as has been widely recommended in various openness agreements [66,67,68,69]. Also, transparency about animal research goes beyond discussions regarding the experiments conducted, the types of research they are, and the species used, reaching how experiments are approved at the regulatory level. For this reason, openness about how animal experiments make it to their implementation stages in labs should also be given the needed attention. This form of openness and transparency needs to stand as proof of the trustworthiness of the regulatory system for animal research. It would be a way of demonstrating to societal stakeholders that proposed experiments go through rigorous evaluations and only legitimate projects are approved and conducted. For this reason, as societal concerns about approval rate are addressed, it might be necessary to consider the need to make available to the general public more information about the proceedings and the criteria used in experiment evaluations.

There seems to be an understanding that there is a need for openness about animal research oversight, knowledge of what goes into project assessments, and access to these assessment documents [70]. It is also recommended that oversight bodies should ‘project transparency in how they evaluated harm and benefit and how they reached their final conclusion’ to assist review by external groups when the need arises, towards informed consensus and broader communication to public on why animals are used in science [6]. Therefore, as a step towards the ideal of ultimate transparency, the publication of the results of AEC decisions—approved and rejected experiments—could be considered. Here, we suggest that there should be reporting of the outcomes of experiment evaluations on online platforms in various jurisdictions, which will be available to the general public. To attain the objectives of the internationalization of animal research ethics, the platform could be an international registry that will be available for all AECs to report details of their evaluations of proposed animal experiments around the world. Specific considerations towards the approval or disapproval of experiments should be provided to inform the public about the decision criteria involved and the evaluation procedures followed in such project evaluations to reach the outcomes. Currently, little or no information is provided on unapproved experiments in the publication of data on animal experiments made available to the public. In this new proposition, more attention could be given to experiments that have not been approved, and AECs could report whether revisions and resubmissions have been demanded and whether the revisions will be made (or are being made) by the researchers or not. When revisions are demanded, what has to be revised should be specified. Revised experiments could later be acceptable for approval; as a result, there should be a link between rejected experiments (for which revisions were demanded) and the revised experiments (which will be accepted later). Also, specifications of experiments that were refused because of an unacceptable harm–benefit ratio (with no possibility of revision or because the scientists did not agree to a revision) should be specified. Making available to the public specific details from project evaluations that make projects unacceptable will show the rigor of animal research project evaluations. It is also a way of being transparent about the evaluation procedure, paving the way for a transparent discussion of HBA in society. To a large extent, it will serve as an avenue for oversight bodies to be responsible for their duties and accountable to society for the decisions they make.

A possible first challenge that the practical implementation of this proposition could face concerns issues regarding confidentiality and intellectual property rights. There might be legitimate fears that ideas might be stolen when details of research projects not yet conducted are made public. To avoid the infringement of intellectual property rights, and to protect the confidentiality of ideas, reports on rejected experiments could be uploaded on this transparency platform after a defined ‘embargo’ time following the decision. In addition to this challenge above, there have been some instances of violent confrontations between members of the public and animal research facilities and the personnel working therein [71,72,73]. Concerning our proposition, we do not advocate for the release of specific details about individuals or their institutions. To ensure the safety and confidentiality of animal researchers, the identities of investigators and the research institutions where these experiments are planned could be anonymized. This is because disclosing the identities of researchers who proposed rejected (or accepted) experiments that could possibly be perceived as unacceptable in the view of the public is an aspect that should be given critical attention since such a move could expose them to several forms of abuse from some groups from the public.

## 9. Conclusions

Oversight criteria, especially HBA, are often criticized for being science-limiting [36,42,43], and as such, the high acceptance rate as a result of joint decisions made by diverse stakeholders in an oversight committee actually appears to show support for science. Also, in discussions around low rejection rates, it is highlighted that there are no criteria regarding how many projects should be rejected [74], and as a matter of fact, we do not think there needs to be one. This is because elaborated criteria for determining the acceptability of experiments should justify the legitimacy of approved experiments, and an experiment that falls short of those criteria is unacceptable and should be rejected. Setting a rate for rejections could lead either to ethically sound projects being rejected or unethical projects being approved. In order to maximize the purpose of HBA as a procedure for animal research regulation and for the sake of public perception of animal research regulation, the current decision procedure needs to ensure that approved projects are unquestionably of scientific and societal value that outweigh the harm caused to animals in experimental procedures.

Animal research is a privilege given by society to scientists to allow animals to be used in research on the condition that only acceptable experiments are conducted. In this paper, we discussed concerns around the high approval rate of animal research oversight. We maintained that the review process needs to be transparent, responsible, and accountable to ensure that even if ethical review of animal experiments always results in positive outcomes, such decisions will always be legitimate and ethically justified. Most importantly, AECs should make sure that before approving experiments, all reasonable efforts are made to implement all legal and ethical requirements to ensure that approved experiments are carried out for the right reasons and animal welfare concerns are well addressed. We propose the publication of the outcomes—positive or negative—of animal experiment evaluations, which will be available to the public. This will not only ensure the transparency of animal research approvals but also further the discussion of HBA in society.

## Figures and Tables

**Table 1:** Possible causes of illegitimate approval and how they can be mitigated.

Possible Causes of High Approval Rate		Mitigating Illegitimate Approvals
**Composition of oversight bodies**	Unequal composition of oversight bodies	A balanced composition of oversight bodies should be ensured
	Scientists dominate the discussions	Committee leaders should ensure balanced contribution from all members
	Lack of consensus leading to a majority-based type of decision-making	Consensus-building should be prioritized over voting-based decision-making
**Focus on harm only**	Excessive attention on revision to modify harm	Attention should be paid to both aspects: benefits and harm
	Tendency to neglect benefit aspects during revision	Avoid excessive modifications for low-benefit experiments/ Conduct another HBA after a request for modification
	Tendency to approve low-benefit experiments	
**Insufficient information on harm**	Approving projects without sufficient information on harm	Project approval should be based on the review of all aspects of harm

## Data Availability

No new data were created or analyzed in this study. Data sharing is not applicable to this article.
